# Adaptation and Validation of the LGBTQIA+ Minority Stress Measure in Spanish Adults

**DOI:** 10.1007/s10508-026-03474-6

**Published:** 2026-07-06

**Authors:** Juan E. Nebot-Garcia, Jaime Barrientos, M. Dolores Gil-Llario, Rafael Ballester-Arnal

**Affiliations:** 1 Faculty of Psychology, Universidad Alberto Hurtado, Santiago, Chile; 2 Faculty of Health Sciences, Department of Basic and Clinical Psychology, and Psychobiology, Universitat Jaume I, Av. Vicent Sos Baynat, s/n, 12071 Castellón de La Plana, Spain; 3 Department of Developmental and Educational Psychology, Universitat de València, Valencia, Spain

**Keywords:** Sexual orientation, Gender identity, Minority stress, Sexual minorities

## Abstract

Lesbian, gay, bisexual, trans, queer, intersex, asexual, and other sexual and gender minorities (LGBTQIA+) face daily stress related to stigma, prejudice, and discrimination, which can negatively impact their mental health. Consequently, having a valid and reliable instrument to assess this experience is essential. This study aimed to adapt and validate the Spanish version of the LGBTQIA+ Minority Stress Measure, originally developed in English for individuals of any sexual orientation and gender identity. A total of 3,254 participants from Spain with an average age of 31.83 years completed the questionnaire. Most participants identified as cisgender (92.4%) and either bisexual (42.7%) or gay men/lesbian (42.2%). Exploratory factor analysis suggested a five-factor structure (identity concealment, rejection anticipation, discrimination events, internalized stigma, and victimization events) with four items per factor and a second-order general factor (LGBTQIA+ Minority Stress). This model was supported by confirmatory factor analysis. The scale showed good internal consistency, adequate convergent validity with anxiety and depression symptoms, and measurement invariance across gender (men, women, nonbinary) and partial invariance across LGBTQIA+ identities (sexual orientation vs. gender identity minorities). These findings support the reliability and validity of the Spanish version of the LGBTQIA+ Minority Stress Measure for assessing minority stress in LGBTQIA+ individuals in Spain. Given the established relationship between minority stress and mental health, this questionnaire may help identify the most affected dimensions and groups, thereby guiding intervention and prevention programs and ultimately contributing to the improvement of LGBTQIA+ individuals’ quality of life.

**Supplementary Information:**

The online version contains supplementary material available at 10.1007/s10508-026-03474-6.

## Introduction

Lesbian, gay, bisexual, trans, queer, intersex, asexual individuals, and other sexual and gender minorities (LGBTQIA+) face a social environment filled with stigma, prejudice, and discrimination against them. This hostile environment requires LGBTQIA+ individuals to exert greater effort to adapt and navigate daily life. This adaptation must be constant and ongoing because the roots of this discrimination lie in well-established social and cultural structures, including family, friends, school, work, communication media, or institutions. It is also important to highlight that, since this situation arises from these social structures, the stigmatized individual has limited agency in avoiding this stress. This extra effort can have significant consequences for the physical and mental health of individuals belonging to a stigmatized social minority, such as LGBTQIA+ individuals. This phenomenon, explained in the minority stress model, was first introduced by Brooks (1981) in the context of lesbian women and later popularized by Meyer (2003), who broadened its scope to include lesbian, gay, and bisexual individuals. It was subsequently extended to encompass transgender individuals (Hendricks & Testa, 2012). Currently, the model aims to explain the mental health of individuals belonging to sexual and gender minorities (Frost & Meyer, 2023).

Specifically, Meyer (2003) conceptualizes minority stress as the situations and processes that LGBTQIA+ individuals are exposed to as a result of belonging to a sexual or gender minority. Meyer identified distal and proximal stressors. Distal minority stressors can be defined as objective stressors, as they do not rely on an individual's own perceptions or appraisals. Rather than depending on personal identification with an assigned minority status, they are based on how society perceives the individual. In other words, regardless of how someone identifies within the spectrum of sexual or gender diversity, if society perceives them as belonging to one of these minority groups, they may still be exposed to discrimination and violence. In contrast, proximal stress processes are more subjective in nature and are closely linked to self-identification as a member of a sexual or gender minority. These identities do not have a single or universal meaning; rather, they vary depending on the social context and each individual's personal experiences. Some individuals may remain vigilant in social situations due to fear of rejection or hostile attitudes (rejection anticipation), hide their identity in some contexts to avoid discrimination or harm (identity concealment), and also internalize societal prejudice and stigma toward sexual and gender diversity, developing negative attitudes toward themselves (internalized stigma).

In this model, Meyer (2003) identified factors that could buffer the negative impact of discrimination and stigma on the mental health of LGBTQIA+ individuals, particularly coping strategies (Aparicio-García et al., 2026) and social support (Li et al., 2023). Subsequently, other variables such as community connectedness (Hoy-Ellis, 2023; Rogers et al., 2021) and pride (Chang et al., 2021; Testa et al., 2015) have also been proposed as potential moderators of this relationship.

Violence based on sexual orientation or gender identity, such as insults, threats, damage to personal property, or physical and sexual assaults, has been associated with greater mental health problems (Sattler & Zeyen, 2021), higher levels of psychological distress (Salerno & Boekeloo, 2022), increased feelings of worry, anxiety, and depression (Flores et al., 2022), and suicidal ideation (Peterson et al., 2021). In school settings, anti-LGBTQIA+ bullying has been linked to lower psychological well-being and higher levels of anxiety and depression (Albaladejo-Blázquez et al., 2019), as well as poorer academic performance and increased absenteeism (Moyano & Sánchez-Fuentes, 2020).

Not only do openly hostile situations impact the mental health of affected individuals, but more subtle forms of discrimination also play a significant role. Scientific literature consistently shows that experiences of discrimination, in which LGBTQIA+ people are marginalized or treated unequally, are associated with poorer mental health, including higher levels of depression, anxiety, and stress (Ngamake et al., 2016; Puckett et al., 2020), as well as lower psychological well-being (Khan et al., 2017). Discrimination can be observed, for example, in the assumption of cisheterosexuality by healthcare professionals, which may lead to misunderstandings, uncomfortable questions, and distress for patients (Enson, 2015; Hayman et al., 2013); in the denial of employment or promotion based on one's identity (Mara et al., 2021); or in the use of incorrect pronouns when referring to someone (Galupo & Resnick, 2016; Guss et al., 2019).

These experiences of discrimination, harassment, and violence faced by LGBTQIA+ individuals are rooted in societal beliefs that portray them as inferior, invalid, or unworthy. Some of these prejudices are easily recognized and rejected by the individuals themselves, while others go unnoticed, are validated, and eventually become integrated into their self-concept, causing them to live their own LGBTQIA+ identity with guilt and shame. This phenomenon is known, among other names, as internalized stigma (Herek et al., 2015). Several studies have demonstrated a close relationship between experiencing discrimination and consequent internalized stigma (Drabish & Theeke, 2022; Feinstein et al., 2012), as well as low acceptance of one's sexual orientation (Woodford et al., 2014) and greater difficulties in identity formation (Berg et al., 2016). Similarly, the presence of internalized stigma had significant implications for the mental health and quality of life of LGBTQIA+ individuals. Internalized stigma has been associated with higher levels of depression (Berg et al., 2016; Feinstein et al., 2012; Gómez et al., 2022; Mohr & Kendra, 2011), suicidal behavior (Skerrett et al., 2016), social anxiety (Feinstein et al., 2012), and psychological distress (Helsen et al., 2022; Puckett et al., 2015). It was also linked to lower life satisfaction (Gómez et al., 2022; Mohr & Kendra, 2011) and lower self-esteem (Austin & Goodman, 2017; Berg et al., 2016; Mohr & Kendra, 2011).

In addition to internalized stigma, experiencing discrimination and violence due to being part of a sexual or gender minority also leads to feelings of anxiety and fear about the possibility of these situations recurring, a phenomenon known as anticipatory rejection or rejection sensitivity (Feinstein, 2020). These feelings can also arise from vicarious experiences, such as witnessing or hearing about similar incidents happening to others (Baams et al., 2020). Thus, LGBTQIA+ individuals may experience anticipatory anxiety about future discriminatory situations and are more likely to perceive ambiguous or low-intensity situations as threatening. Additionally, they may have a greater tendency to remember instances of rejection, prolonging negative emotions over time. Several studies have demonstrated the association between discrimination and rejection sensitivity (Dyar et al., 2016; Feinstein et al., 2012; Mereish et al., 2019), as well as the connection between rejection sensitivity and internalized stigma (Dyar et al., 2016; Feinstein et al., 2012, 2017; Glon et al., 2020). Rejection sensitivity in LGBTQIA+ individuals has also been linked to mental health issues (Helsen et al., 2022), such as suicidal ideation and behavior (Mereish et al., 2019), depression, and anxiety (Dyar et al., 2016; Feinstein et al., 2017; Glon et al., 2020), and specifically, social anxiety (Feinstein et al., 2012).

Given the internalization of prejudice and stigma, along with the anticipation of future rejections, it is common for many in the LGBTQIA+ population to hide their true identity. In certain high-risk contexts, concealment can even be an adaptive strategy. However, widespread concealment may hinder access to social support and limit the development of trust and meaningful connections with others (Huang & Chan, 2022). Several studies have shown that concealing one's sexual orientation or gender identity is closely linked to internalized stigma (Flynn & Bhambhani, 2021; Pistella et al., 2016) and rejection sensitivity (Dyar et al., 2016). Concealment was related to higher levels of depression and generalized anxiety (Feinstein et al., 2020), greater psychological distress (Craig et al., 2024), and lower psychological well-being (Brownfield & Brown, 2022), life satisfaction (Flynn & Bhambhani, 2021), and positive affect and overall happiness (Bejakovich & Flett, 2018).

In light of the relationship between these minority stress factors and mental health, it is highly necessary to be able to assess them adequately to intervene effectively. Many studies have used a specific measure to assess a particular aspect of minority stress, such as discrimination and/or aggression (Testa et al., 2015), internalized stigma (Bockting et al., 2020), rejection anticipation (Pachankis et al., 2008), and identity concealment or disclosure (Jackson & Mohr, 2016). However, few scales comprehensively assess several or all dimensions of minority stress. For example, the Lesbian, Gay, and Bisexual Identity Scale (Mohr & Kendra, 2011) evaluates concealment motivation and internalized homonegativity, along with other dimensions. However, it does not cover important aspects proposed by Meyer (2003), such as discrimination or rejection anticipation. Additionally, this scale is limited to lesbian, gay men, or bisexual individuals. On the other hand, the Daily Heterosexist Experiences Questionnaire (Balsam et al., 2013) is accessible to anyone with a minority sexual orientation or gender identity. But despite its broader applicability, it omits some crucial dimensions like internalized stigma and rejection anticipation. Additionally, it includes some very specific dimensions, such as those related to concerns about HIV. This aspect can be controversial, as it appears to assume that LGBTQIA+ individuals have contact with people who have HIV, exemplified by the item “Worrying about your friends who have HIV.” Historically, the early years of the HIV epidemic were closely associated with certain segments of the LGBTQIA+ population, particularly due to the high number of infections among gay men (Handlovsky et al., 2024) and trans women (Poteat et al., 2014, 2016), as well as the caregiving roles assumed by lesbian women (Klassen, 2021), among others. However, other LGBTQIA+ groups have had a less direct association with HIV. Moreover, in Spain, approximately 16.6–29.0% of men and 70.7–79.9% of women acquired HIV through male–female sexual intercourse (Del Romero et al., 2023; Unidad de Vigilancia de VIH & ITS y Hepatitis, 2024). Therefore, it is no longer appropriate to associate or limit HIV exclusively to the LGBTQIA+ community. Given the persistence of HIV-related stigma (Schweitzer et al., 2023), maintaining this association may contribute to further stigma and prejudice against LGBTQIA+ individuals (Rice et al., 2022) and may also reinforce a false sense of safety and a low perception of HIV risk among the heterosexual population (Tan et al., 2021; Tieosapjaroen et al., 2023). In contrast, the Gender Minority Stress and Resilience Measure (Testa et al., 2015) covers all the dimensions proposed by Meyer (2003) but is specifically designed for individuals with a minority gender identity.

Meanwhile, the LGBT Minority Stress Measure (Outland, 2016) comprehensively captures the full range of stressors outlined in Meyer’s (2003) framework, assessing six dimensions related to proximal and distal stress (identity concealment, microaggressions, rejection anticipation, discrimination events, internalized stigma, and victimization) and one moderator dimension (community connectedness). Additionally, it can be answered by individuals with both a minority sexual orientation and a minority gender identity. The Minority Stress Scale Among Italian Gay and Bisexual Men (Norcini Pala et al., 2017) assesses expectations of discrimination, sexual orientation concealment, internalized homophobia, verbal and physical aggressions, and discrimination and social exclusion. However, it is tailored for gay and bisexual men residing in Italy. Lastly, the Sexual Minority Adolescent Stress Inventory (Schrager et al., 2018) measures some relevant dimensions, such as internalized homonegativity, concealment, and social marginalization. Although it is designed for the broader LGBTQIA+ community, its focus remains on adolescents. In Spain, the only available adaptation is the Daily Heterosexist Experiences Questionnaire (Balsam et al., 2013). In their Spanish version, Ronzón-Tirado et al. (2023) removed some dimensions from the original, such as the one related to HIV. Nevertheless, internalized stigma and rejection anticipation remain unassessed at this questionnaire.

Based on all this information, we consider the LGBT Minority Stress Measure (Outland, 2016) to be the most appropriate tool for providing a comprehensive and inclusive assessment of minority stress in our context. However, this measure presents some limitations. For example, the original version of the questionnaire does not include an exploratory factor analysis (EFA), and the proposed model does not assess a general second-order factor. Therefore, our study aims to adapt and validate the LGBT Minority Stress Measure (Outland, 2016) among individuals with any minority sexual orientation or gender identity within the Spanish population. For this adaptation, only the six dimensions related to distal and proximal stressors were considered, excluding Community Connectedness, as it is regarded as a moderating variable rather than a core component of minority stress (Frost et al., 2022; Rogers et al., 2021).

This study also aims to conduct an EFA to examine whether the factor structure proposed in the original version is replicated, followed by a confirmatory factor analysis (CFA) testing the six dimensions along with a general second-order factor (LGBTQIA+ Minority Stress). Furthermore, the psychometric properties, convergent validity, and internal consistency of the Spanish version will be evaluated. Finally, given that several studies have shown that men exhibited higher levels of concealment (Bachmann & Gooch, 2018), discrimination (Moyano & Sánchez-Fuentes, 2020), and internalized stigma (Bregman et al., 2013; Paul et al., 2014), an invariance analysis will be conducted to confirm that the scale performs equally across genders. Similarly, trans individuals showed higher levels of discrimination, harassment, and violence compared to cisgender lesbian, gay, and bisexual individuals (Bauerband et al., 2019; Bayrakdar & King, 2023; Kattari et al., 2016), as well as greater vigilance in situations involving discrimination (Bauerband et al., 2019). Additionally, other studies have found that trans women report higher levels of internalized stigma than cisgender women with a minority sexual orientation (Logie et al., 2020), and that bisexual trans individuals experience greater minority stress than bisexual cisgender individuals (Katz-Wise et al., 2017). Due to these differences, an additional invariance analysis will be conducted to ensure that the scale functions consistently regardless of whether individuals identify with a minority gender identity or are cisgender with a minority sexual orientation.

The main hypothesis is that the Spanish version of the LGBTQIA+ Minority Stress Measure will replicate the original six-factor structure proposed by Outland (2016), with a good model fit and robust psychometric properties, including a general second-order factor. The second hypothesis posited that the instrument will also demonstrate measurement invariance across different gender identities and LGBTQIA+ groups.

## Method

### Participants

This study included a total of 3,254 participants residing in Spain. The average age of the sample was 31.83 years (*SD* = 11.68), with ages ranging from 18 to 72 years. As detailed in Table 1, the majority of participants identified as bisexual (42.7%) or gay men/lesbian (42.2%), held Spanish citizenship (93.5%), identified as atheist or agnostic (74.5%), leaned towards a progressive political ideology (73.4%), and had attained a university degree or higher (59%). While most participants identified as cisgender (92.4%), the sample also included individuals with other gender identities, including trans men (1.5%), trans women (1%), and people who do not identify with the traditional categories of man or woman, that is, nonbinary individuals (5%). Participants were from various provinces across Spain, with Madrid (17.1%), Barcelona (15.6%), Valencia (7.4%), and Sevilla (3.8%) being the most represented.

**Table 1:** Main sociodemographic characteristics of participants

Variables	Total	EFA	CFA
	(*n* = 3254)	(*n* = 1627)	(*n* = 1627)
	*M (SD)*	*M (SD)*	*M (SD)*
Age (in years)	31.83 (11.68)	31.62 (11.73)	32.04 (11.63)
	n (%)	n (%)	n (%)
*Gender identity*			
Cis men	1526 (46.9)	742 (45.6)	784 (48.2)
Cis women	1481 (45.5)	758 (46.6)	723 (44.4)
Trans men	49 (1.5)	27 (1.7)	22 (1.4)
Trans women	34 (1)	15 (0.9)	19 (1.2)
Nonbinary gender	164 (5)	85 (5.2)	79 (4.9)
*Sexual orientation*			
Heterosexual	22 (0.7)	9 (0.6)	13 (0.8)
Gay/Lesbian	1372 (42.2)	679 (41.7)	693 (42.6)
Bisexual	1388 (42.7)	712 (43.8)	676 (41.5)
Pansexual	241 (7.4)	112 (6.9)	129 (7.9)
Asexual	126 (3.9)	63 (3.9)	63 (3.9)
Other	105 (3.2)	52 (3.2)	53 (3.3)
*Nationality*			
Spanish	3043 (93.5)	1521 (93.5)	1522 (93.5)
Spanish conationality	17 (0.5)	8 (0.5)	9 (0.6)
Foreigner	194 (6)	98 (6)	96 (5.9)
*Religious beliefs*			
Practicing believer	159 (4.9)	90 (5.5)	69 (4.2)
Nonpracticing believer	670 (20.6)	322 (19.8)	348 (21.4)
Atheist or agnostic	2425 (74.5)	1215 (74.7)	1210 (74.4)
*Political ideology*			
Conservative	112 (3.4)	55 (3.4)	57 (3.5)
Center	390 (12)	192 (11.8)	198 (12.2)
Progressive	2389 (73.4)	1201 (73.8)	1188 (73)
Indifferent	363 (11.2)	179 (11)	184 (11.3)
*Level of education*			
Without studies	3 (0.1)	2 (0.1)	1 (0.1)
Primary	55 (1.7)	27 (1.7)	27 (1.7)
Secondary	585 (17.9)	285 (17.5)	300 (18.4)
Vocational training	695 (21.3)	330 (20.3)	363 (22.3)
Diploma/Bachelor/Degree	1236 (37.9)	623 (38.3)	609 (37.4)
Master/Doctorate	689 (21.1)	360 (22.1)	327 (20.1)

### Measures

This research is part of the comprehensive Safo Project, which focuses on assessing affective-sexual diversity in Spain, alongside examining mental health challenges and related factors among people with varying sexual orientations and gender identities. In this particular study, apart from collecting sociodemographic information, one questionnaire and two subscales from another questionnaire were considered.

All participants completed our version of the LGBT Minority Stress Measure (Outland, 2016), which we renamed the Spanish version of the LGBTQIA+ Minority Stress Measure, available in the Appendix. This initial Spanish version consisted of 41 Likert-type items, distributed across six dimensions: identity concealment, microaggressions, rejection anticipation, discrimination events, internalized stigma, and victimization events. Respondents answered each item on a scale ranging either from 1 ("*never happens*") to 5 ("*happens all the time*") or from 1 ("*strongly disagree*") to 5 ("*strongly agree*"). The total score ranged from 41 to 205, with higher scores indicating greater minority stress.

In addition, participants completed the Depression and Anxiety subscales of the Symptom Assessment-45 Questionnaire (Davison et al., 1997). The Spanish version was used in this study (Sandín et al., 2008). Participants were asked to indicate how much each symptom had been present during the past week. Each dimension consists of five items rated on a 5-point Likert scale, ranging from 0 (*'Not at all'*) to 4 (*'Very much or extremely'*). In the present study, both the Depression scale (α_ordinal_ = .92) and the Anxiety scale (α_ordinal_ = .91) showed excellent internal consistency.

### Procedure

#### Translating and Adapting the Scale

In line with the "committee approach" for translating questionnaires (Maneesriwongul & Dixon, 2004), three experts in psychology specializing in sexual and gender diversity independently translated the LGBT Minority Stress Measure (Outland, 2016) into Spanish. These independent translations were then combined and carefully reviewed by the experts. They compared the merged version with the original to ensure accuracy and consistency. Any required adjustments were made to guarantee that both the original and the translated versions conveyed the same meaning. Additionally, following the recommendations of the American Psychological Association (2020), which considers the term "LGBT" outdated, we changed all mentions of LGBT individuals in the items of the original scale to a more inclusive form, such as LGBTQIA+. We also aimed to maintain gender-neutral wording in the translation. Once all items had been translated and reviewed, we proceeded to analyze their content in detail, eliminating a total of 4 items (see Table 2). These items were removed because they addressed specific aspects that could not be answered by the LGBTQIA+ community as a whole. For example, “I avoid talking about my romantic life because I do not want others to know I am LGBTQIA+” focused on sexual and romantic attraction (i.e., sexual orientation), whereas “When organizations or activities are divided by gender, I feel out of place because I am LGBTQIA+” focused more on gender identity.

**Table 2:** Flow of item refinement leading to the final version

Identity concealment
**1. I avoid telling people certain aspects of my life that might indicate I am LGBTQIA+. **
2. I avoid talking about my romantic life because I do not want others to know I am LGBTQIA+.^a^
**3. I change my gestures or the way I speak because I do not want others to think I am LGBTQIA+. **
4. I do not bring dates to social events because I do not want others to know I am LGBTQIA+.^a^
**5. I do not express disagreement when I hear anti-LGBTQIA+ comments because I do not want others to assume I am LGBTQIA+. **
**6. I limit what I share on social media, or what can be seen, because I do not want others to know I am LGBTQIA+. **
Microaggressions
7. I have difficulty finding people I can identify with in TV shows, movies, books, music, etc.^b^
8. I have been accused of “flaunting” my LGBTQIA+ identity.^b^
9. I am expected to educate non-LGBTQIA+ people about LGBTQIA+ issues.^b^
10. I have been told that I am not really LGBTQIA+, but that I am confused or just seeking attention.^b^
11. In school, I was not taught about the important contributions of LGBTQIA+ people throughout history.^b^
12. I have been introduced by others as “my LGBTQIA+ friend” or “the LGBTQIA+ one.”^b^
13. People assume that my sexual orientation or gender is something different from what it actually is.^b^
14. People have relabeled my identity or referred to me using a name or pronouns different from how I identify.^b^
15. I have been introduced to a potential date or friend with the expectation that we would get along just because the other person was also LGBTQIA+.^b^
16. I have overheard people make anti-LGBTQIA+ comments.^b^
17. I feel uncomfortable using public restrooms or locker rooms because I am LGBTQIA+.^b^
18. When organizations or activities are divided by gender, I feel out of place because I am LGBTQIA+.^a^
19. I have been accused of being too defensive or politically correct when talking about LGBTQIA+ issues with someone who is not LGBTQIA+.^b^
Rejection Anticipation
**20. When I meet someone new, I worry that deep down they may dislike me because I am LGBTQIA+. **
21. When I go out in public with my partner, I fear that people will treat us unkindly because I am LGBTQIA+.^a^
**22. I stay on guard and alert because something bad might happen to me because I am LGBTQIA+. **
**23. I brace myself to be treated disrespectfully because I am LGBTQIA+. **
**24. I expect that others will not accept me because I am LGBTQIA+. **
25. I worry about what will happen if people find out I am LGBTQIA+.^c^
Discrimination Events
**26. I have been excluded from an organization (e.g. a religious group, sports team, etc.) because I am LGBTQIA+. **
**27. Healthcare providers have pressured me into receiving unnecessary services or have denied me services because I am LGBTQIA+. **
**28. I have been denied housing or been mistreated in my place of residence (e.g. college dorm, home owner’s association, etc.) because I am LGBTQIA+.**
**29. I have received poor service at a business because I am LGBTQIA+.**
30. I am forced to consider my LGBTQIA+ identity when I think about politics.^c^
31. I have been treated unfairly by supervisors or teachers because I am LGBTQIA+.^d^
Internalized Stigma
**32. If I were offered the chance to be someone who is not LGBTQIA+, I would accept the opportunity.**
33. I wish I were not LGBTQIA+.^d^
**34. I feel that being LGBTQIA+ is a personal flaw in me.**
35. I feel that me being LGBTQIA+ must have been a mistake of fate/nature/God/etc.^c^
**36. I wonder why I am not “normal” like everyone else.**
**37. I envy people who are not LGBTQIA+. **
38. I have tried to stop being LGBTQIA+.^e^
Victimization Events
**39. I have been verbally harassed or called names because I am LGBTQIA+. **
40. I have received unwanted sexual attention or been asked inappropriate questions about my sexual life because I am LGBTQIA+.^c^
**41. I have been physically attacked because I am LGBTQIA+. **
**42. Others have damaged my personal property because I am LGBTQIA+. **
43. I have endured unwanted sexual contact because I am LGBTQIA+.^d^
44. Others have threatened to harm me because I am LGBTQIA+.^e^
**45. I have been bullied because I am LGBTQIA+. **

*Note*. The English wording shown corresponds to our adapted version of the instrument, not to the original source. Items retained in the final version are indicated in bold. ^a^Removed prior to the exploratory factor analysis due to its wording focused on sexual orientation or gender identity. ^b^Dimension removed in the first round due to its low contribution to the common construct intended to be measured. ^c^Items removed in the second round due to issues with factorial loadings. ^d^Items removed in the third round due to issues with factorial loadings. ^e^Items removed in the fourth round to ensure an equal number of four items per factor

#### Recruiting for the Exploratory Factor Analysis/Confirmatory Factor Analysis Study

From November 2019 to November 2020, a series of advertisements were posted on Facebook and Instagram in Spain, inviting individuals to take part in a study on sexuality. Upon clicking these ads, users were directed to an initial screen where they were informed that participation in the study was anonymous, voluntary, and confidential. Participants did not receive any compensation for their involvement. They were also required to give informed consent before proceeding to complete an online Qualtrics questionnaire. This convenience sampling method garnered 1,961 responses. Additionally, between December 2019 and April 2020, direct contact was made with various Spanish LGBTQIA+ associations and organizations. These groups were introduced to the research and asked to disseminate it through their social networks and contacts, resulting in an additional 1,600 responses. Altogether, these methods yielded 3,561 responses.

After applying inclusion criteria (being over 18 years old and residing in Spain), 73 responses from underage individuals and 201 responses from individuals living outside Spain were excluded, leaving 3,287 valid responses. Furthermore, 33 responses were removed due to participants not indicating their sexual orientation or gender identity. 

### Statistical Analyses

Initially, sociodemographic data (including gender identity, sexual orientation, nationality, religious beliefs, political ideology, level of education, and province of residence) were analyzed descriptively using the SPSS statistical package (version 29.0).

#### Exploratory Factor Analysis

The *psych* package (Revelle, 2025) and the *EFAtools* package (Steiner & Grieder, 2020), both in the R software environment (Posit Team, 2025), were used to conduct the EFAs. First, to determine whether the data met the assumptions necessary for conducting factor analysis, it was verified that the Kaiser–Meyer–Olkin (KMO) index exceeded .80 (Kaiser, 1974) and that Bartlett’s test of sphericity was statistically significant (Bartlett, 1950). Then, the number of factors was estimated using a parallel analysis based on a polychoric correlation matrix, employing principal axis factoring as the extraction method. Finally, an EFA with oblimin rotation was performed to identify the internal structure of the scale.

To determine which items remained in the final version and to which factor they belonged, we considered factor loadings above .30 (Lloret-Segura et al., 2014), although some authors adopt a stricter threshold of .40 (Howard, 2016). Several additional criteria were also applied to refine the item pool. For instance, items with communalities below .40 were removed because they indicate that the item shares little variance with the rest (Costello & Osborne, 2005). Items loading above .30 on multiple factors were also eliminated unless the difference between cross-loadings exceeded .20 (Howard, 2016), although other authors recommend differences of .30 or .40 (Lloret-Segura et al., 2014). Problematic items presenting Heywood cases—namely, factor loadings greater than 1.0, an impossible outcome—were likewise removed (Costello & Osborne, 2005). Finally, we ensured that all subscales contained the same number of items to facilitate the use and scoring of the self-administered questionnaire, improve psychometric performance, and avoid overlap across dimensions (Durao et al., 2019; Hadžibajramović et al., 2022).

#### Confirmatory Factor Analysis

A CFA was conducted using the *lavaan* package (Rosseel, 2012) within the R software environment (Posit Team, 2025), employing the Weighted Least Square Mean and Variance adjusted (WLSMV) estimator to evaluate the fit of the proposed factorial structure. In accordance with methodological recommendations (Izquierdo et al., 2014), the EFA and CFA were performed on independent subsamples. These subsamples were randomly generated using SPSS (version 29.0). Specifically, each participant was assigned a random decimal value between 0.00 and 1.00. Those with values less than or equal to 0.49 were allocated to the EFA subsample, while those with values ranging from 0.50 to 1.00 were assigned to the CFA subsample. The characteristics of both subsamples are presented in Table 1.

Various indices were used to evaluate the model fit of the CFA. Given the tendency of the chi-square statistic to be influenced by large sample sizes and to be significant even with reasonably good model fit (Bentler & Bonett, 1980), additional fit indices were employed: Root Mean Square Error of Approximation (RMSEA), Comparative Fit Index (CFI), Tucker-Lewis Index (TLI), and Standardized Root Mean Square Residual (SRMR). According to criteria set by Bagozzi and Yi (2011), excellent fit was defined as CFI and TLI ≥ .95, and RMSEA and SRMR ≤ .05. Alternatively, less stringent criteria proposed by Hooper et al. (2008) considered CFI and TLI ≥ .90, RMSEA ≤ .08, and SRMR ≤ .10 as acceptable.

#### Item Descriptive Statistics and Internal Consistency

Corrected item-total correlations were calculated using the full sample of 3,254 individuals in SPSS (version 29.0) to assess item reliability, with values above .30 generally considered acceptable (Stefana et al., 2025). Descriptive statistics were also obtained for the items, including skewness and kurtosis. Following the guidelines proposed by Weston and Gore (2006), univariate normality was assumed when skewness was less than 3.0 and kurtosis was less than 10.0. Additionally, to examine group differences in minority stress levels, we calculated the mean scores and 95% confidence intervals for each of the five minority stress dimensions by gender identity and by sexual orientation. Lastly, Ordinal Cronbach's alpha (α_ordinal_) was computed for both subfactors and the general factor using the *psych* R package (Revelle, 2025). Following guidelines established by Hunsley and Mash (2008), alpha values between .70 and .79 were deemed acceptable, those between .80 and .89 were considered good, and values equal to or greater than .90 were classified as excellent.

#### Structural Invariance

The structural invariance analysis was conducted using the *lavaan* package (Rosseel, 2012) and the *semTools* package (Jorgensen et al., 2025) in R (Posit Team, 2025). The aim was to investigate potential variations in the structure of the Spanish version of the LGBTQIA+ Minority Stress Measure based on participants' gender and LGBTQIA+ identity. In the gender analysis, comparisons were conducted between cisgender and transgender men, cisgender and transgender women, as well as nonbinary individuals. Regarding LGBTQIA+ identity, structural differences were examined between gender identity minorities of any sexual orientation (including trans men, trans women, and nonbinary individuals) and cisgender individuals with minority sexual orientations (such as lesbian, gay, bisexual, pansexual, asexual, and others). Therefore, the aim was to compare individuals with a minority gender identity to those with a minority sexual orientation. Individuals who identified as both gender and sexual orientation minorities were categorized under gender identity minorities. This decision was made in order to prioritize gender identity minority, given its higher association with elevated levels of minority stress (Katz-Wise et al., 2017; Logie et al., 2020).

Considering the substantial differences in group sizes and their potential impact on invariance results (Chen, 2007), efforts were made to balance the samples prior to conducting the invariance analyses. To achieve this, two new random data variables were generated, distinct from the one used to split the sample for the EFA and CFA. Although the number of nonbinary participants was 164, the size of the other groups was slightly increased, following the recommendations by MacCallum et al. (1999) regarding appropriate sample sizes for robust factor analysis results. Therefore, the 200 men (cisgender and transgender) and 200 women (cisgender and transgender) with the lowest random values were selected. This procedure was repeated to select the next 200 men and 200 women with the subsequent lowest random values, thereby creating two independent subsamples. In each subsample, measurement invariance was tested simultaneously across the three groups (men, women, and nonbinary participants). This allowed us to replicate the invariance analysis with separate samples in order to assess the robustness and stability of the results, given that the limited sample size may compromise their reliability (Chen, 2007; MacCallum et al., 1999). This same procedure was applied to select two subsamples of 247 cisgender individuals with minority sexual orientations, in order to compare them with individuals with a minority gender identity. Each invariance model's fit was assessed by comparing nested models (△) using RMSEA, CFI, and SRMR indices. A change of ≥ .01 in CFI, ≥ .01 in RMSEA, or ≥ .03 in SRMR indicates a significant decrease in model fit when testing for measurement invariance (Chen, 2007).

#### Convergent Validity

To assess the convergent validity, Pearson correlations were conducted between the different subfactors, as well as between the subfactors and the general factor with the Anxiety and Depression dimensions of the Symptom Assessment-45 Questionnaire (Davison et al., 1997; Sandín et al., 2008). According to Cohen (1988), correlations were classified as small when ranging from 0.10 to 0.29, moderate from 0.30 to 0.49, and large when equal to or above 0.50.

To examine whether the associations between minority stress dimensions and mental health outcomes differed across groups, we computed separate correlation matrices for LGBTQIA+ identity groups (gender identity minority vs. sexual orientation minority) and for gender groups (men, women, and nonbinary).

## Results

### Exploratory Factor Analysis

The structure of the data was adequate for factor analysis, as indicated by a KMO index greater than .80 (0.91), and a statistically significant Bartlett’s test of sphericity (*χ*^*2*^ = 46,512.39, *df* = 820, *p* < .001). Parallel analysis based on exploratory factor analysis (EFA) suggested the retention of eight factors.

We initially conducted an EFA specifying eight factors and observed that, in some dimensions such as Microaggressions and Internalized Stigma, several items loaded on two different factors, diverging from the theoretical structure. This solution accounted for 58.8% of the total variance. We then tested a six-factor solution, which aligned more closely with our theoretical expectations. In this model, previously scattered dimensions were more clearly represented within a single factor, and the solution explained 55.6% of the total variance. However, as shown in Table S1 (Supplementary Material), some items from different dimensions still loaded on unexpected factors or showed very low communalities (below .30), prompting us to refine the factor structure. We observed that 9 out of the 12 items in the Microaggressions dimension showed communalities below .40, with the highest communality reaching only .55. This indicates that most items in this factor shared a very limited amount of common variance with the rest of the scale, suggesting a weak contribution to the underlying construct. Therefore, we decided to remove the Microaggressions factor from the final version of the questionnaire. The flow of item removals across the different phases of the EFA is detailed in Table 2.

After excluding this factor, we conducted a new parallel analysis. In this case, five factors were suggested, with satisfactory values for both the KMO measure (0.91) and Bartlett’s test of sphericity (*χ*^*2*^ = 37,029.37, *df* = 406, *p* < .001). Based on the results of the new EFA, we proceeded to refine the dimensions that presented issues. To avoid abrupt modifications, we opted to remove one item per dimension in a stepwise manner. Four items were removed due to problematic factor loadings (Table S2, Supplementary Material). Item 25 (Rejection Anticipation) and Item 40 (Victimization Events) showed cross-loadings on multiple factors, while Item 30 (Discrimination Events) and Item 35 (Internalized Stigma) loaded primarily on factors that lacked theoretical justification or did not align with their intended dimensions.

A new exploratory factor analysis was conducted based on the revised structure, revealing improved alignment across dimensions. However, further refinement was necessary for some of them (Table S3, Supplementary Material). Item 31 (Discrimination Events) and Item 43 (Victimization Events) were removed, as they exhibited higher loadings on factors other than their theoretically assigned ones. Additionally, Item 33 (Internalized Stigma) was excluded due to a factor loading exceeding 1.0, which likely reflected extreme multicollinearity or redundancy with other Items.

Once these items had been removed, the new factor analysis showed a good fit, with all items exhibiting communalities above .40 and explaining 65.6% of the total variance. However, while most dimensions contained four items, the Internalized Stigma and Victimization Events dimensions each included five. To achieve consistency across factors, one item from each of these two dimensions was removed so that all dimensions contained an equal number of items. Given that all remaining items demonstrated satisfactory psychometric properties, the items eliminated were those whose wording or content appeared less representative of the construct they intended to measure. Consequently, Item 38 from Internalized Stigma and Item 44 from Victimization Events were excluded (Table S4, Supplementary Material).

The final model was reanalyzed using parallel analysis, and the results suggested a five-factor structure, with satisfactory values for the KMO measure (0.88) and Bartlett’s test of sphericity (*χ*^*2*^ = 21,572.01, *df* = 190, *p* < .001). The proposed factor solution accounted for 65.9% of the total variance, and the factor loadings and their distribution are presented in Table 3. All items showed communalities above .40. Only two items (41 and 42 from the Victimization Events dimension) exhibited cross-loadings above .30 on two factors; however, the difference between these loadings exceeded .30, with the highest loading occurring on their intended factor.

**Table 3:** Exploratory factor analysis with rotated components matrix and eigenvalue for 5-factor model and communality

	Factorial loadings					
Items	Factor 1	Factor 2	Factor 3	Factor 4	Factor 5	Communality
Item 1	− 0.119	0.071	0.045	**0.736**	− 0.056	0.607
Item 3	0.277	0.044	0.048	**0.639**	0.002	0.575
Item 5	0.069	− 0.011	0.112	**0.579**	0.072	0.443
Item 6	− 0.030	0.004	− 0.011	**0.822**	0.020	0.668
Item 20	0.069	**0.535**	0.092	0.235	− 0.012	0.507
Item 22	0.067	**0.838**	0.003	− 0.039	− 0.005	0.746
Item 23	− 0.011	**0.937**	0.003	− 0.063	0.038	0.865
Item 24	− 0.045	**0.795**	0.004	0.179	0.009	0.709
Item 26	0.252	0.174	0.011	0.025	**0.465**	0.532
Item 27	− 0.001	0.205	0.086	− 0.114	**0.568**	0.456
Item 28	0.053	− 0.034	0.041	0.087	**0.761**	0.640
Item 29	0.191	0.230	− 0.008	− 0.090	**0.516**	0.559
Item 32	0.071	− 0.003	**0.780**	0.010	− 0.094	0.615
Item 34	− 0.144	− 0.075	**0.787**	0.108	0.196	0.739
Item 36	0.006	− 0.025	**0.845**	0.003	− 0.001	0.706
Item 37	0.082	0.116	**0.836**	− 0.047	− 0.062	0.744
Item 39	**0.853**	0.095	0.032	− 0.004	− 0.059	0.782
Item 41	**0.625**	0.022	− 0.015	− 0.016	**0.315**	0.699
Item 42	**0.694**	− 0.018	0.005	0.006	**0.305**	0.772
Item 45	**0.915**	0.000	0.028	0.023	− 0.056	0.805
Eigenvalue	3.093	2.951	2.848	2.256	2.024	

*Note*. Factor loadings greater than .30 are shown in bold

### Confirmatory Factor Analysis

**Fig. 1:**
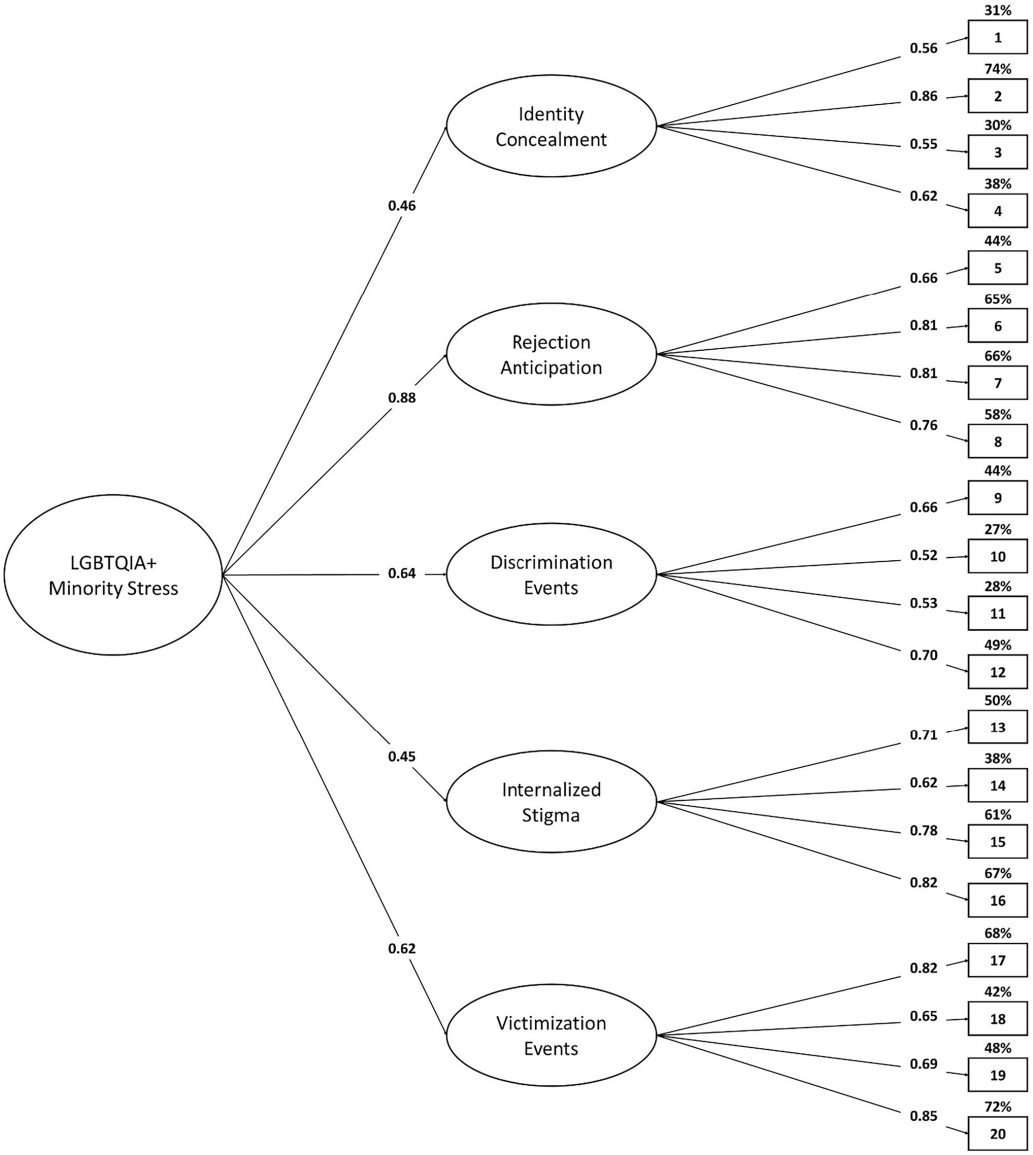
Confirmatory factor analysis for the Spanish version of the LGBTQIA+ Minority Stress Measure. Note. R^2^ is expressed as a percentage outside the main endogenous variables, represented by boxes. All endogenous variables are significant at *p* < .001. The coefficients are reported in standardized format. To enhance interpretation, error terms are not included.

The structure of the Spanish version of the LGBTQIA+ Minority Stress Measure was examined through a CFA. The model demonstrated acceptable fit across all indices (*χ*^*2*^ = 1245.19; *df* = 165; *χ*^*2*^*/df* = 7.55; *p* < .001; RMSEA = .058; CFI = .916; TLI = .904; SRMR = .078). Figure 1 presents the five-factor model with a second-order general factor derived from this version.

### Item Descriptive Statistics and Internal Consistency

Furthermore, descriptive statistics about the items and the corrected correlations between the items and their respective subfactors were calculated. As shown in Table 4, all items exhibited correlation values above .40, ranging from .42 to .76. These correlations were statistically significant at *p* < .001. On the other hand, although most items display skewness and kurtosis values within the acceptable range for univariate normality, some items, particularly those in the Discrimination Events subfactor, show elevated levels of skewness and kurtosis. Further information regarding mean differences in the five minority stress dimensions by gender identity and sexual orientation is provided in Figures S1 and S2 (Supplementary Material), which display the corresponding means and 95% confidence intervals.

**Table 4:** Descriptive statistics and reliability indexes for items and factors

Factors and items	Range	*M (SD)*	IQR (25–50-75)	SK	K	*rit*
**Identity concealment**	4–20	6.92 (3.03)	NA	1.34	1.72	NA
Item 1	1–5	2.33 (1.20)	1–2-3	0.55	-0.65	.558
Item 3	1–5	1.51 (0.93)	1–1-2	1.96	3.32	.522
Item 5	1–5	1.37 (0.80)	1–1-1	2.49	6.31	.439
Item 6	1–5	1.71 (1.10)	1–1-2	1.48	1.22	.606
**Rejection anticipation**	4–20	7.65 (3.62)	NA	1.06	0.69	NA
Item 20	1–5	1.75 (1.04)	1–1-2	1.34	1.01	.558
Item 22	1–5	1.99 (1.14)	1–2-3	0.98	0.07	.713
Item 23	1–5	1.89 (1.07)	1–2-3	1.10	0.44	.769
Item 24	1–5	2.01 (1.11)	1–2-3	0.93	0.03	.712
**Discrimination events**	4–20	4.77 (1.60)	NA	3.36	15.65	NA
Item 26	1–5	1.25 (0.68)	1–1-1	3.19	11.05	.434
Item 27	1–5	1.15 (0.53)	1–1-1	4.22	20.34	.455
Item 28	1–5	1.10 (0.43)	1–1-1	5.43	34.24	.420
Item 29	1–5	1.26 (0.60)	1–1-1	2.64	7.69	.526
**Internalized stigma**	4–20	5.86 (2.99)	NA	2.00	3.94	NA
Item 32	1–5	1.65 (1.10)	1–1-2	1.58	1.50	.617
Item 34	1–5	1.22 (0.66)	1–1-1	3.55	13.42	.561
Item 36	1–5	1.48 (0.97)	1–1-1	2.08	3.40	.637
Item 37	1–5	1.51 (1.00)	1–1-2	1.94	2.78	.685
**Victimization events**	4–20	6.08 (2.84)	NA	1.85	3.65	NA
Item 39	1–5	1.91 (1.07)	1–2-3	0.99	0.15	.698
Item 41	1–5	1.25 (0.65)	1–1-1	3.03	9.92	.632
Item 42	1–5	1.23 (0.63)	1–1-1	3.27	11.50	.650
Item 45	1–5	1.69 (1.06)	1–1-2	1.50	1.40	.738
**Total**	20–100	31.29 (9.48)	NA	1.36	2.34	NA

*Note.* IQR = Interquartile Range; SK = Skewness; K = Kurtosis; *rit* = corrected item–total correlation within subfactor; NA = Not Applicable. All correlations were statistically significative at *p* < .001

The internal consistency of the general factor was excellent (α_ordinal_ = .91), as was that of the subfactors Internalized Stigma (α_ordinal_ = .90) and Victimization Events (α_ordinal_ = .92). Rejection Anticipation (α_ordinal_ = .89), Discrimination Events (α_ordinal_ = .84), and Identity Concealment (α_ordinal_ = .82) also demonstrated good internal consistency.

### Structural Invariance

The measurement invariance of the Spanish version was first evaluated across gender groups (men, women, and nonbinary). As shown in Table 5, both samples yielded moderately acceptable fit indices for configural invariance, although TLI values were slightly below the recommended threshold (.89 and .87), and the CFI in the second sample was also somewhat low (.89). When proceeding with the subsequent invariance analyses, no substantial declines in model fit were observed when testing for metric, scalar, and residual invariance in either sample (△RMSEA < .01; △CFI < .01; △SRMR < .03).

**Table 5:** Multigroup confirmatory factor analysis by gender and LGBTQIA+ identity

	χ^2^	*df*	χ^2^/*df*	*p*	RMSEA (90% CI)	CFI	TLI	SRMR	Comparisons	△ RMSEA	△ CFI	△ SRMR
**Gender**												
*Sample 1*												
Configural invariance	616.289	330	1.87	< .001	.061 [.054, .069]	.909	.895	.084	NA			
Metric invariance	544.855	349	1.56	< .001	.061 [.051, .071]	.904	.896	.089	C vs. M	< .001	.005	.004
Scalar invariance	572.532	363	1.58	< .001	.062 [.052, .072]	.897	.893	.090	S vs. M	.001	.007	.002
Residual invariance	598.033	383	1.56	< .001	.062 [.052, .071]	.892	.893	.097	R vs. S	< .001	.005	.006
*Sample 2*												
Configural invariance	692.408	330	2.10	< .001	.066 [.059, .073]	.893	.877	.090	NA			
Metric invariance	568.874	349	1.63	< .001	.064 [.054, .073]	.894	.885	.093	C vs. M	.002	.001	.003
Scalar invariance	598.393	363	1.65	< .001	.065 [.055, .074]	.887	.881	.095	S vs. M	.001	.008	.002
Residual invariance	629.070	383	1.64	< .001	.065 [.056, .074]	.879	.880	.103	R vs. S	< .001	.008	.008
**LGBTQIA+ Identity**												
*Sample 1*												
Configural invariance	644.189	330	1.95	< .001	.066 [.058, .073]	.912	.899	.091	NA			
Metric invariance	543.537	349	1.58	< .001	.064 [.053, .074]	.913	.905	.096	C vs. M	.002	.001	.005
Scalar invariance	567.636	363	1.56	< .001	.064 [.054, .074]	.908	.904	.097	S vs. M	< .001	.005	.001
Residual invariance	607.868	383	1.59	< .001	.066 [.056, .076]	.897	.898	.109	R vs. S	.002	.011	.012
*Sample 2*												
Configural invariance	632.008	330	1.92	< .001	.070 [.062, .078]	.890	.873	.100	NA			
Metric invariance	580.019	349	1.66	< .001	.071 [.060, .081]	.882	.871	.103	C vs. M	< .001	.008	.003
Scalar invariance	607.102	363	1.67	< .001	.071 [.061, .081]	.874	.869	.105	S vs. M	.001	.007	.002
Residual invariance	638.621	383	1.67	< .001	.071 [.062, .081]	.867	.868	.115	R vs. S	< .001	.008	.010

*Note.* χ^2^ = Satorra-Bentler chi-square; *df* = degrees of freedom; *p* = general model significance; χ^2^/*df* = normed chi-square; RMSEA = root mean square error of approximation; CFI = comparative fit index; TLI = Tucker-Lewis index; SRMR = standardized root mean square residual; NA = not available; C = configural; M = metric; S = scalar; R = residual

Additionally, measurement invariance was examined across LGBTQIA+ identity groups (gender identity minority vs. sexual orientation minority). The results for configural invariance differed between the two samples (Table 5). Sample 1 showed acceptable fit, with only a slightly low TLI (.89). In contrast, Sample 2 presented somewhat low values for both CFI (.89) and TLI (.87), and the SRMR was at the upper limit of acceptability (.10). When proceeding with the subsequent analyses, no significant declines in model fit were observed when testing metric and scalar invariance across LGBTQIA+ identity in either sample (△RMSEA < .01; △CFI < .01; △SRMR < .03). However, for residual invariance, Sample 2 showed acceptable values across all indices, while Sample 1 also demonstrated generally acceptable fit, although the change in CFI slightly exceeded the recommended threshold (△CFI = .01).

### Convergent Validity

As can be seen in Table 6, all subfactors of the scale displayed statistically significant positive correlations with one another. Notably, moderate correlations were found between Identity Concealment and both Rejection Anticipation and Internalized Stigma, as well as between Rejection Anticipation and both Discrimination Events and Victimization Events. A large correlation was also observed between Discrimination Events and Victimization Events. On the other hand, the different subfactors showed positive correlations with Depression and Anxiety, although these were of small effect size. Similarly, the total scale showed statistically significant positive correlations with Depression (*r* = .27) and Anxiety (*r* = .25). Additional information on these correlations across LGBTQIA+ identity groups (gender identity minority vs. sexual orientation minority) and gender groups (men, women, and nonbinary) is provided in Table S5 (Supplementary Material).

**Table 6:** Correlations between the subfactors of the scale and symptoms of anxiety and depression

	1	2	3	4	5
**Total**					
1. Identity Concealment					
2. Rejection Anticipation	**.335**				
3. Discrimination Events	.109	**.386**			
4. Internalized Stigma	**.418**	.295	.174		
5. Victimization Events	.114	**.455**	**.529**	.194	
Depression	.157	.245	.121	.225	.138
Anxiety	.139	.246	.154	.149	.151

*Note*. All correlations were significant at *p* < .001. Medium and large correlations are shown in bold

## Discussion

The LGBT Minority Stress Measure (Outland, 2016) was selected due to its comprehensive assessment of all dimensions proposed by Meyer (2003) and its applicability to individuals with diverse minority sexual orientations and gender identities. The present study aimed to translate the scale into Spanish and evaluate its psychometric properties in a Spanish sample. The final factorial structure demonstrated good model fit and satisfactory psychometric properties, consisting of five dimensions with four items each, and a general second-order factor. The structure and functioning of the scale did not vary across gender groups, and partial measurement invariance was found across LGBTQIA+ identity groups. Nonetheless, further research is needed to explore this aspect more thoroughly.

During the adaptation process, the Community Connectedness dimension was excluded, as it is considered a mediating variable between minority stress and mental health rather than a direct component of minority stress itself (Frost et al., 2022; Rogers et al., 2021). Additionally, contrary to the main hypothesis, the Microaggressions dimension was removed during the EFA due to inadequate factor loadings. These difficulties may be related to the inherent complexity in defining and measuring microaggressions (Mekawi & Todd, 2021). Previous research has also highlighted variations in the perception and detection of microaggressions based on gender and sexual orientation (Banks & Landau, 2019; Basford et al., 2014). Furthermore, LGBTQIA+ individuals may experience different types of microaggressions depending on their specific sexual orientation or gender identity (Nadal et al., 2016); for example, lesbian women may not be exposed to the same forms of microaggressions as bisexual women or transgender individuals. Finally, several items from other dimensions were also excluded because of inadequate factor loadings or wording issues.

The final Spanish version of the LGBTQIA+ Minority Stress Measure consisted of 20 items, distributed across five dimensions (Identity Concealment, Rejection Anticipation, Discrimination Events, Internalized Stigma, and Victimization Events), with four items each, along with a general second-order factor (LGBTQIA+ Minority Stress). This structure demonstrated good model fit and overall reliability. The general factor reached excellent levels of reliability, and all dimensions showed good to excellent internal consistency. Regarding the corrected item-total correlations, all items exceeded the threshold of .40, indicating good levels of item reliability (Stefana et al., 2025). Although most items showed acceptable values for assuming univariate normality, some exhibited high levels of skewness and kurtosis. Such values are common in scales assessing traumatic events related to aggression and discrimination, which tend to occur infrequently (Buelga et al., 2019; Mahmood et al., 2023; Velez & Moradi, 2012). In Spain, public attitudes toward homosexuality are generally positive, with 89% of the population expressing acceptance (Pew Research Center, 2020). This widespread social acceptance may partly account for the lower frequencies observed in some of the experiences assessed. However, these figures do not imply the absence of discrimination or violence based on sexual orientation or gender identity. According to a European survey (European Union Agency for Fundamental Rights, 2020), 11% of LGBTI individuals in Spain reported experiencing discrimination while looking for a job in the past 12 months, and 20% reported workplace discrimination due to their sexual orientation or gender identity. Additionally, 8% of Spanish LGBTI individuals aged 15 and older reported having suffered physical and/or sexual assaults in the previous five years, and 41% reported experiencing harassment in the past 12 months due to being LGBTI. Further evidence from the Spanish Ministry of the Interior (2024) indicates that in 2023, a total of 522 hate crimes related to sexual orientation or gender identity were officially reported to the police. Although some of these figures may seem relatively low, they demonstrate that such experiences are still present in Spanish society. Therefore, they were retained due to their theoretical relevance to the construct being measured. Nevertheless, these deviations from normality are unlikely to have significantly affected model fit, as the WLSMV estimator and a polychoric correlation matrix were employed, both of which are recommended for producing robust results when analyzing 5-point Likert-type items with non-normal distributions (Finney & DiStefano, 2013; Flora & Flake, 2017; Li, 2016).

Despite evidence that variables related to minority stress are experienced differently depending on gender (Bachmann & Gooch, 2018; Bregman et al., 2013; Demant et al., 2018; Moyano & Sánchez-Fuentes, 2020; Paul et al., 2014) and LGBTQIA+ identity (Bauerband et al., 2019; Bayrakdar & King, 2023; Jones & Hillier, 2013; Kattari et al., 2016; Logie et al., 2020), and in line with the second hypothesis, invariance analyses indicated that the structure of the Spanish version of the LGBTQIA+ Minority Stress Measure remains consistent across gender identities (male, female, or nonbinary) in both subsamples, and across LGBTQIA+ identities (gender identity minority vs. sexual orientation minority), with full invariance observed in one subsample and partial invariance in the other. It should also be noted that some indices for the configural invariance model were slightly below optimal levels. However, some authors have argued that the same cutoff values should not be expected across models with varying degrees of complexity (Cheung & Rensvold, 2002). Considering the complexity of the present model, which included five dimensions with only four items each and a second-order general factor, a more flexible approach was adopted and subsequent invariance testing was carried out. These findings of invariance support the usefulness of this measure compared to other similar questionnaires that focus on only one segment of the LGBTQIA+ community (Mohr & Kendra, 2011; Norcini Pala et al., 2017; Testa et al., 2015). Nevertheless, these results should be interpreted with caution, and further research with larger and balanced samples is needed to obtain more robust conclusions.

Regarding the correlations among the different subfactors of the scale, all of them showed statistically significant associations with one another, and each displayed medium or large correlations with one or more subfactors. This supports the theoretical validity of the scale and the potential existence of a common general factor. Moreover, none of the correlations were excessively high, indicating that the subfactors are not redundant but rather complementary. Additionally, all dimensions and the general factor showed statistically significant correlations with both the Anxiety and Depression dimensions, although the effect sizes were small. These values are comparable to those reported in previous studies examining associations between sexual and gender minority stress variables and mental health outcomes (Craig et al., 2024; Gómez et al., 2022; Puckett et al., 2020; Sattler & Zeyen, 2021).

Although the results were significant, it is important to note some limitations of the present study. First, this questionnaire assesses minority stress experiences across the entire LGBTQIA+ community, allowing us to compare these experiences among different LGBTQIA+ groups and identify which groups are most affected in each dimension. However, as a broad and comprehensive questionnaire designed for the entire LGBTQIA+ community, it lacks the specificity and distinct characteristics of each individual group within the community. Therefore, to better understand the specific experiences of a group, it would be advisable to use questionnaires tailored to that population or conduct individual interviews. Second, since the measures rely on participants' retrospective reports, there may be potential memory biases. The nature and characteristics of the items could also have led to social desirability biases in the responses due to the study of stigmatized groups, although the anonymous administration of the scale helps to reduce this risk. Moreover, using self-report measures exclusively may introduce some degree of same-method covariance inflation; however, this is a common limitation in questionnaire-based research and does not substantially compromise the interpretation of the findings. Third, while convergent validity was examined through correlations with depression and anxiety, these variables represent psychological sequelae rather than constructs that directly overlap with minority stress processes. Including such proximal measures would have provided a more comprehensive assessment of convergent validity. Future research could benefit from incorporating measures that capture conceptually closer minority stress constructs. Fourth, another methodological limitation relates to the format in which the items were presented. Specifically, the items appeared in an ordered sequence, with the four items of each subfactor grouped consecutively. This structure may have facilitated response consistency or demand characteristics, potentially inflating within-factor correlations. Future adaptations of the scale could randomize item order to minimize response patterns and improve psychometric rigor. Fifth, although the translation process followed the committee approach (Maneesriwongul & Dixon, 2004), the study did not conduct a formal back-translation. This may introduce a minor source of translation bias, and future adaptations would benefit from including this additional step. Finally, it is necessary to include more individuals from certain underrepresented groups, such as asexual or nonbinary individuals, to obtain more conclusive and representative results.

In conclusion, the results of this study indicate that the Spanish version of the LGBTQIA+ Minority Stress Measure is a useful, reliable, valid, and appropriate tool for assessing the stressful experiences faced by LGBTQIA+ individuals in Spain. These experiences include identity concealment, rejection anticipation, discrimination events, internalized stigma, and victimization events. Given the relationship between minority stress and mental health (Craig et al., 2024; Feinstein et al., 2020; Gómez et al., 2022; Helsen et al., 2022; Petruzzella et al., 2019; Puckett et al., 2020; Sherman et al., 2020), it is important to highlight that this tool enables the analysis of all the experiences proposed by Meyer (2003) in individuals of any sexual orientation and gender identity. This, in turn, will facilitate the study of mental health in the LGBTQIA+ population, allowing for the identification of more vulnerable groups and the dimensions most affected within each group. Such insights will help focus intervention and prevention programs on these issues, ultimately improving the quality of life for LGBTQIA+ individuals. Additionally, providing this Spanish version broadens the potential reach to nearly 500 million Spanish speakers (Instituto Cervantes, 2024), offering a foundation for future validation efforts in diverse Spanish-speaking contexts.

## Electronic supplementary material

Below is the link to the electronic supplementary material.

Supplementary file1 (DOCX 84 KB)

## Data Availability

The data from this research are not in any public repository. However, to contribute to transparency in science, the data can be shared upon justified request to the corresponding author.

## Appendix

### Versión Española de la Medida de Estrés de las Minorías LGBTQIA+ (Spanish version of the LGBTQIA+ Minority Stress Measure)

*Instrucciones*: Por favor, lee cada enunciado con atención y luego indica con qué frecuencia ocurre la situación descrita en tu vida o tu grado de acuerdo o desacuerdo con el enunciado.

*Corrección*: Hacer el sumatorio de los ítems de cada dimensión para obtener la puntuación de cada dimensión y el total.

**Table:**

**Ocultación de la identidad** 1 (“*nunca me ocurre*”) – 5 (“*me ocurre siempre*”)	
1. Evito contar a la gente ciertos aspectos de mi vida que podrían significar que soy LGBTQIA+	1—2—3—4—5
2. Cambio mis gestos o forma de hablar porque no quiero que otras personas piensen que soy LGBTQIA+	1—2—3—4—5
3. No muestro mi desacuerdo cuando escucho discursos anti-LGBTQIA+ porque no quiero que otras personas asuman que soy LGBTQIA+	1—2—3—4—5
4. Limito lo que comparto en redes sociales, o lo que puede verse, porque no quiero que otras personas sepan que soy LGBTQIA+	1—2—3—4—5
**Rechazo anticipatorio** 1 (“*nunca me ocurre*”) – 5 (“*me ocurre siempre*”)	
5. Cuando conozco a alguien nuevo, me preocupa que en el fondo no le guste por ser LGBTQIA+	1—2—3—4—5
6. Estoy en guardia y alerta porque algo malo puede ocurrirme por ser LGBTQIA+	1—2—3—4—5
7. Me preparo para recibir un trato irrespetuoso por ser LGBTQIA+	1—2—3—4—5
8. Anticipo que otras personas no me aceptarán por ser LGBTQIA+	1—2—3—4—5
**Eventos discriminatorios** 1 (“*nunca me ocurre*”) – 5 (“*me ocurre siempre*”)	
9. Me han excluido de una organización (p.e. un grupo religioso, un equipo deportivo, etc.) por ser LGBTQIA+	1—2—3—4—5
10. Personal sanitario me ha presionado para recibir servicios innecesarios o me ha denegado servicios por ser LGBTQIA+	1—2—3—4—5
11. Me han negado la vivienda o me han discriminado en mi lugar de residencia (por ejemplo, residencia universitaria, comunidad de vecinos, etc.) por ser LGBTQIA+	1—2—3—4—5
12. He recibido una mala atención en un establecimiento por ser LGBTQIA+	1—2—3—4—5
**Estigma internalizado** 1 (“*muy en desacuerdo*”) – 5 (“*muy de acuerdo*”)	
13. Si me ofrecieran la oportunidad de ser alguien que no es LGBTQIA+ , aceptaría la oportunidad	1—2—3—4—5
14. Siento que ser LGBTQIA+ es un defecto personal mío	1—2—3—4—5
15. Me pregunto por qué no soy "normal" como el resto de personas	1—2—3—4—5
16. Envidio a la gente que no es LGBTQIA+	1—2—3—4—5
**Eventos de victimización** 1 (“*nunca me ocurre*”) – 5 (“*me ocurre siempre*”)	
17. Me han acosado verbalmente o me han puesto motes por ser LGBTQIA+	1—2—3—4—5
18. Me han agredido físicamente por ser LGBTQIA+	1—2—3—4—5
19. Han dañado mis propiedades personales por ser LGBTQIA+	1—2—3—4—5
20. He sufrido bullying por ser LGBTQIA+	1—2—3—4—5
