# Supplementary material for: Adaptation and Validation of the LGBTQIA+ Minority Stress Measure in Spanish Adults
Source: Arch Sex Behav. 2026 Jul 6;55(5):1989–2007. doi: 10.1007/s10508-026-03474-6 (PMC13427841; doi:10.1007/s10508-026-03474-6)

**Table S1**

Rotated components matrix for the 6-factor solution (initial model)

|  | Factorial loadings | | | | | |  |
| --- | --- | --- | --- | --- | --- | --- | --- |
| Items | Factor 1 | Factor 2 | Factor 3 | Factor 4 | Factor 5 | Factor 6 | Communality |
| Item 1 | -0.060 | -0.007 | 0.019 | 0.020 | **0.815** | -0.001 | 0.681 |
| Item 3 | **0.359** | 0.095 | -0.159 | -0.012 | **0.578** | 0.107 | 0.544 |
| Item 5 | 0.149 | 0.021 | -0.042 | -0.026 | **0.522** | 0.224 | 0.412 |
| Item 6 | 0.044 | -0.064 | 0.041 | 0.055 | **0.772** | -0.008 | 0.607 |
| Item 7* | -0.130 | 0.136 | **0.426** | 0.043 | 0.125 | 0.003 | 0.240 |
| Item 8* | 0.232 | 0.140 | **0.494** | -0.081 | -0.202 | 0.143 | 0.557 |
| Item 9* | 0.144 | 0.079 | **0.514** | 0.016 | -0.077 | -0.170 | 0.422 |
| Item 10* | 0.009 | -0.078 | **0.627** | -0.026 | 0.078 | 0.063 | 0.369 |
| Item 11* | -0.004 | 0.099 | **0.358** | 0.031 | 0.050 | -0.314 | 0.243 |
| Item 12* | **0.437** | 0.080 | 0.213 | 0.009 | -0.128 | -0.018 | 0.382 |
| Item 13* | -0.047 | -0.003 | **0.468** | 0.114 | 0.234 | -0.168 | 0.270 |
| Item 14* | 0.287 | 0.091 | 0.275 | -0.061 | 0.020 | 0.141 | 0.307 |
| Item 15* | **0.425** | 0.114 | 0.146 | 0.040 | -0.162 | 0.003 | 0.352 |
| Item 16* | 0.012 | 0.122 | **0.375** | -0.002 | 0.151 | -0.098 | 0.230 |
| Item 17* | **0.317** | 0.258 | 0.148 | 0.032 | -0.002 | 0.055 | 0.364 |
| Item 19* | 0.019 | 0.191 | **0.567** | -0.099 | -0.027 | -0.013 | 0.472 |
| Item 20 | 0.065 | **0.444** | 0.170 | 0.154 | 0.262 | -0.060 | 0.543 |
| Item 22 | 0.094 | **0.859** | -0.053 | 0.006 | -0.046 | -0.024 | 0.763 |
| Item 23 | 0.044 | **0.908** | 0.002 | -0.012 | -0.068 | 0.033 | 0.838 |
| Item 24 | -0.025 | **0.736** | 0.102 | 0.003 | 0.219 | 0.008 | 0.741 |
| Item 25 | -0.161 | **0.400** | 0.056 | 0.107 | **0.565** | 0.049 | 0.694 |
| Item 26 | **0.504** | 0.102 | 0.185 | -0.063 | 0.012 | 0.189 | 0.498 |
| Item 27 | 0.281 | 0.105 | **0.300** | -0.016 | -0.121 | 0.242 | 0.385 |
| Item 28 | **0.503** | -0.084 | 0.161 | -0.110 | 0.048 | **0.359** | 0.454 |
| Item 29 | **0.469** | 0.112 | 0.285 | -0.051 | -0.094 | 0.154 | 0.544 |
| Item 30 | 0.039 | 0.250 | **0.359** | 0.039 | -0.107 | **-0.303** | 0.373 |
| Item 31 | **0.555** | 0.110 | 0.201 | -0.051 | -0.007 | 0.128 | 0.561 |
| Item 32 | 0.063 | -0.024 | -0.038 | **0.932** | -0.013 | -0.033 | 0.838 |
| Item 33 | 0.001 | -0.008 | -0.003 | **0.935** | 0.010 | 0.074 | 0.956 |
| Item 34 | -0.048 | 0.046 | -0.036 | **0.342** | 0.106 | **0.654** | 0.865 |
| Item 35 | 0.011 | 0.026 | -0.072 | 0.254 | 0.077 | **0.704** | 0.812 |
| Item 36 | -0.022 | 0.011 | 0.110 | **0.414** | 0.101 | **0.455** | 0.662 |
| Item 37 | 0.063 | 0.124 | 0.050 | **0.629** | 0.049 | 0.184 | 0.685 |
| Item 38 | -0.062 | 0.075 | 0.172 | **0.380** | 0.163 | 0.249 | 0.463 |
| Item 39 | **0.767** | 0.123 | -0.025 | 0.128 | -0.013 | -0.102 | 0.700 |
| Item 40 | **0.383** | 0.119 | **0.380** | 0.100 | -0.083 | -0.149 | 0.524 |
| Item 41 | **0.835** | 0.007 | 0.003 | 0.010 | -0.018 | 0.042 | 0.722 |
| Item 42 | **0.922** | -0.035 | -0.031 | 0.004 | 0.034 | 0.020 | 0.805 |
| Item 43 | **0.482** | -0.111 | **0.327** | 0.079 | 0.035 | 0.019 | 0.418 |
| Item 44 | **0.850** | 0.064 | 0.022 | -0.027 | 0.028 | -0.030 | 0.782 |
| Item 45 | **0.836** | 0.058 | -0.090 | 0.101 | 0.033 | -0.109 | 0.701 |

*Note*. Items marked with an asterisk were deleted. Factor loadings greater than .300 are shown in bold.

**Table S2**

Rotated components matrix for the 5-factor solution after item removal: Step 1

|  | Factorial loadings | | | | |  |
| --- | --- | --- | --- | --- | --- | --- |
| Items | Factor 1 | Factor 2 | Factor 3 | Factor 4 | Factor 5 | Communality |
| Item 1 | -0.089 | 0.050 | 0.005 | **0.786** | -0.004 | 0.647 |
| Item 3 | **0.320** | 0.000 | 0.017 | **0.646** | 0.034 | 0.550 |
| Item 5 | 0.140 | -0.020 | -0.017 | **0.570** | 0.180 | 0.429 |
| Item 6 | 0.023 | -0.006 | 0.025 | **0.773** | -0.014 | 0.607 |
| Item 20 | 0.090 | **0.542** | 0.125 | 0.238 | -0.052 | 0.534 |
| Item 22 | 0.099 | **0.801** | 0.035 | -0.039 | -0.046 | 0.721 |
| Item 23 | 0.060 | **0.883** | 0.014 | -0.072 | 0.016 | 0.814 |
| Item 24 | -0.019 | **0.809** | -0.005 | 0.187 | 0.013 | 0.758 |
| Item 25* | -0.178 | **0.475** | 0.094 | **0.525** | 0.059 | 0.683 |
| Item 26 | **0.550** | 0.178 | -0.082 | -0.016 | 0.213 | 0.497 |
| Item 27 | **0.344** | 0.243 | -0.058 | -0.175 | **0.304** | 0.368 |
| Item 28 | **0.544** | -0.027 | -0.131 | 0.032 | **0.389** | 0.460 |
| Item 29 | **0.531** | 0.237 | -0.092 | -0.135 | 0.201 | 0.517 |
| Item 30* | 0.112 | **0.443** | -0.050 | -0.182 | -0.207 | 0.295 |
| Item 31 | **0.603** | 0.192 | -0.076 | -0.041 | 0.160 | 0.555 |
| Item 32 | 0.054 | -0.019 | **0.948** | -0.016 | -0.057 | 0.846 |
| Item 33 | -0.005 | 0.016 | **0.934** | 0.007 | 0.066 | 0.948 |
| Item 34 | -0.043 | -0.013 | **0.389** | 0.140 | **0.611** | 0.862 |
| Item 35* | 0.019 | -0.072 | **0.320** | 0.130 | **0.633** | 0.791 |
| Item 36 | 0.008 | 0.044 | **0.428** | 0.093 | **0.444** | 0.655 |
| Item 37 | 0.074 | 0.153 | **0.638** | 0.037 | 0.178 | 0.687 |
| Item 38 | -0.034 | 0.166 | **0.369** | 0.141 | 0.252 | 0.446 |
| Item 39 | **0.770** | 0.092 | 0.137 | 0.009 | -0.130 | 0.688 |
| Item 40* | **0.456** | **0.321** | 0.029 | -0.158 | -0.077 | 0.452 |
| Item 41 | **0.854** | -0.021 | 0.015 | -0.011 | 0.037 | 0.728 |
| Item 42 | **0.929** | -0.070 | 0.014 | 0.041 | 0.010 | 0.812 |
| Item 43 | **0.540** | 0.075 | 0.017 | -0.041 | 0.091 | 0.367 |
| Item 44 | **0.868** | 0.051 | -0.019 | 0.035 | -0.050 | 0.782 |
| Item 45 | **0.824** | -0.002 | 0.117 | 0.057 | -0.138 | 0.686 |

*Note*. Items marked with an asterisk were deleted. Factor loadings greater than .300 are shown in bold.

**Table S3**

Rotated components matrix for the 5-factor solution after item removal: Step 2

|  | Factorial loadings | | | | |  |
| --- | --- | --- | --- | --- | --- | --- |
| Items | Factor 1 | Factor 2 | Factor 3 | Factor 4 | Factor 5 | Communality |
| Item 1 | -0.122 | 0.040 | 0.070 | **0.733** | -0.051 | 0.596 |
| Item 3 | **0.301** | 0.022 | 0.041 | **0.667** | -0.051 | 0.584 |
| Item 5 | 0.075 | 0.062 | -0.008 | **0.609** | 0.067 | 0.441 |
| Item 6 | -0.045 | 0.021 | 0.012 | **0.785** | 0.007 | 0.637 |
| Item 20 | 0.058 | 0.104 | **0.538** | 0.232 | -0.004 | 0.511 |
| Item 22 | 0.076 | -0.002 | **0.839** | -0.035 | -0.028 | 0.745 |
| Item 23 | -0.009 | 0.006 | **0.939** | -0.068 | 0.032 | 0.864 |
| Item 24 | -0.054 | 0.011 | **0.798** | 0.173 | 0.015 | 0.712 |
| Item 26 | **0.304** | -0.006 | 0.167 | 0.034 | **0.423** | 0.531 |
| Item 27 | 0.016 | 0.067 | 0.210 | -0.122 | **0.585** | 0.478 |
| Item 28 | 0.152 | 0.039 | -0.052 | 0.081 | **0.691** | 0.611 |
| Item 29 | 0.251 | -0.021 | 0.217 | -0.089 | **0.489** | 0.575 |
| Item 31* | **0.403** | -0.016 | 0.175 | -0.017 | **0.362** | 0.574 |
| Item 32 | 0.072 | **0.941** | -0.006 | -0.067 | -0.098 | 0.824 |
| Item 33* | 0.008 | **1.002** | -0.002 | -0.039 | -0.047 | 0.953 |
| Item 34 | -0.149 | **0.694** | -0.065 | 0.183 | 0.268 | 0.706 |
| Item 36 | -0.026 | **0.644** | -0.012 | 0.150 | 0.133 | 0.573 |
| Item 37 | 0.061 | **0.735** | 0.121 | 0.044 | 0.031 | 0.679 |
| Item 38 | -0.049 | **0.499** | 0.108 | 0.175 | 0.068 | 0.424 |
| Item 39 | **0.849** | 0.060 | 0.100 | -0.002 | -0.115 | 0.750 |
| Item 41 | **0.728** | 0.024 | -0.007 | -0.031 | 0.207 | 0.723 |
| Item 42 | **0.806** | 0.009 | -0.052 | 0.019 | 0.195 | 0.804 |
| Item 43* | **0.303** | 0.052 | 0.054 | -0.030 | **0.385** | 0.403 |
| Item 44 | **0.776** | -0.054 | 0.077 | 0.009 | 0.128 | 0.777 |
| Item 45 | **0.900** | 0.040 | 0.010 | 0.035 | -0.105 | 0.754 |

*Note*. Items marked with an asterisk were deleted. Factor loadings greater than .300 are shown in bold.

**Table S4**

Rotated components matrix for the 5-factor solution after item removal: Step 3

|  | Factorial loadings | | | | |  |
| --- | --- | --- | --- | --- | --- | --- |
| Items | Factor 1 | Factor 2 | Factor 3 | Factor 4 | Factor 5 | Communality |
| Item 1 | -0.121 | 0.040 | 0.067 | **0.736** | -0.045 | 0.601 |
| Item 3 | 0.291 | 0.047 | 0.038 | **0.642** | -0.024 | 0.576 |
| Item 5 | 0.073 | 0.106 | -0.010 | **0.583** | 0.065 | 0.441 |
| Item 6 | -0.041 | -0.006 | 0.009 | **0.820** | 0.023 | 0.670 |
| Item 20 | 0.067 | 0.097 | **0.536** | 0.231 | -0.019 | 0.509 |
| Item 22 | 0.071 | 0.003 | **0.837** | -0.038 | -0.014 | 0.747 |
| Item 23 | -0.009 | 0.000 | **0.937** | -0.060 | 0.037 | 0.861 |
| Item 24 | -0.048 | 0.013 | **0.797** | 0.174 | 0.009 | 0.712 |
| Item 26 | **0.305** | 0.029 | 0.180 | 0.022 | **0.413** | 0.527 |
| Item 27 | 0.029 | 0.091 | 0.227 | -0.115 | **0.545** | 0.454 |
| Item 28 | 0.139 | 0.055 | -0.025 | 0.090 | **0.703** | 0.633 |
| Item 29 | 0.256 | 0.004 | 0.235 | -0.091 | **0.469** | 0.565 |
| Item 32 | 0.074 | **0.767** | -0.019 | 0.014 | -0.086 | 0.600 |
| Item 34 | -0.140 | **0.792** | -0.076 | 0.105 | 0.208 | 0.741 |
| Item 36 | 0.003 | **0.856** | -0.035 | -0.007 | 0.010 | 0.714 |
| Item 37 | 0.086 | **0.847** | 0.101 | -0.055 | -0.064 | 0.752 |
| Item 38* | -0.018 | **0.651** | 0.094 | 0.059 | -0.043 | 0.498 |
| Item 39 | **0.858** | 0.047 | 0.090 | -0.009 | -0.118 | 0.758 |
| Item 41 | **0.713** | -0.016 | 0.003 | -0.005 | 0.237 | 0.721 |
| Item 42 | **0.793** | 0.010 | -0.044 | 0.015 | 0.219 | 0.806 |
| Item 44* | **0.765** | -0.049 | 0.084 | 0.002 | 0.151 | 0.779 |
| Item 45 | **0.911** | 0.045 | 0.000 | 0.017 | -0.114 | 0.766 |

*Note*. Items marked with an asterisk were deleted. Factor loadings greater than .300 are shown in bold.

**Table S5**

*Correlations between the subfactors of the scale and symptoms of anxiety and depression by LGBTQIA+ identity and gender*

|  | 1 | 2 | 3 | 4 | 5 |
| --- | --- | --- | --- | --- | --- |
| **Sexual orientation minority** |  |  |  |  |  |
| 1. Identity Concealment |  |  |  |  |  |
| 2. Rejection Anticipation | **.342**** |  |  |  |  |
| 3. Discrimination Events | .120** | **.377**** |  |  |  |
| 4. Internalized Stigma | **.416**** | .291** | .162** |  |  |
| 5. Victimization Events | .131** | **.449**** | **.511**** | .197** |  |
| Depression | .156** | .239** | .093** | .228** | .119** |
| Anxiety | .134** | .233** | .124** | .153** | .129** |
| **Gender identity minority** |  |  |  |  |  |
| 1. Identity Concealment |  |  |  |  |  |
| 2. Rejection Anticipation | **.364**** |  |  |  |  |
| 3. Discrimination Events | .088 | **.344**** |  |  |  |
| 4. Internalized Stigma | **.467**** | **.328**** | .262** |  |  |
| 5. Victimization Events | < .001 | **.386**** | **.577**** | .143* |  |
| Depression | .219** | .185** | .249** | .163* | .202** |
| Anxiety | .256** | .233** | .276** | .079 | .209** |
| **Cis and trans men** |  |  |  |  |  |
| 1. Identity Concealment |  |  |  |  |  |
| 2. Rejection Anticipation | **.309**** |  |  |  |  |
| 3. Discrimination Events | .100** | **.385**** |  |  |  |
| 4. Internalized Stigma | **.421**** | .295** | .165** |  |  |
| 5. Victimization Events | .059* | **.451**** | **.499**** | .159** |  |
| Depression | .242** | **.349**** | .164** | **.321**** | .215** |
| Anxiety | .211** | **.339**** | .196** | .248** | .228** |
| **Cis and trans women** |  |  |  |  |  |
| 1. Identity Concealment |  |  |  |  |  |
| 2. Rejection Anticipation | **.360**** |  |  |  |  |
| 3. Discrimination Events | .086** | **.349**** |  |  |  |
| 4. Internalized Stigma | **.348**** | .258** | .118** |  |  |
| 5. Victimization Events | .108** | **.431**** | **.541**** | .111** |  |
| Depression | .124** | .167** | .054* | .194** | .133** |
| Anxiety | .148** | .192** | .106** | .138** | .186** |
| **Nonbinary gender** |  |  |  |  |  |
| 1. Identity Concealment |  |  |  |  |  |
| 2. Rejection Anticipation | **.326**** |  |  |  |  |
| 3. Discrimination Events | .170* | **.380**** |  |  |  |
| 4. Internalized Stigma | **.397**** | **.331**** | **.340**** |  |  |
| 5. Victimization Events | -.004 | **.379**** | **.603**** | .217** |  |
| Depression | .221** | .155 | .269** | .166* | .173* |
| Anxiety | .252** | .208** | **.305**** | .110 | .176* |

*Note*. **p* < .05, ***p* < .01, ****p* < .001. Medium and large correlations are shown in bold.

**Figure S1**

Mean scores and 95% confidence intervals on five minority stress dimensions by gender identity


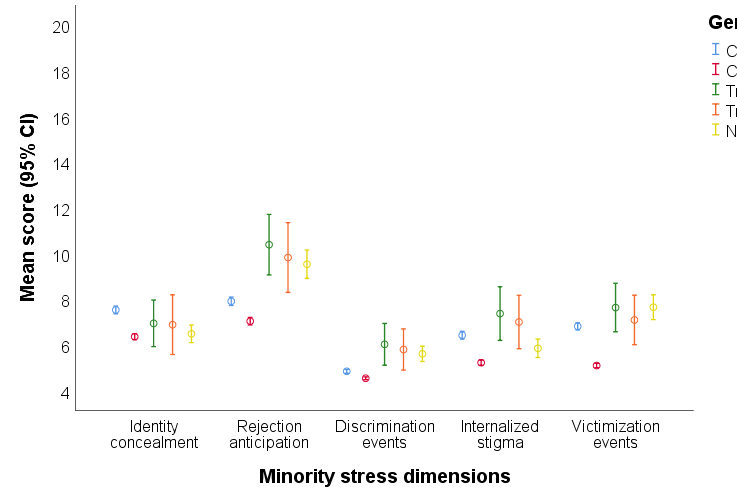


**Figure S2**

Mean scores and 95% confidence intervals on five minority stress dimensions by sexual orientation


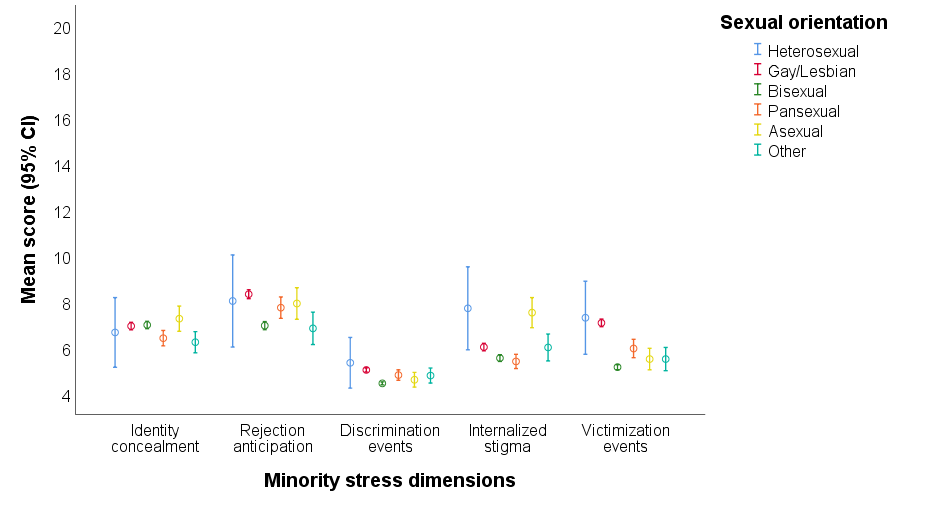

Supplement: Supplementary file 1 — Supplementary file1 (DOCX 84 KB) [file 10508_2026_3474_MOESM1_ESM.docx]
